# Supplementary material for: SyNDI: synchronous network data integration framework
Source: BMC Bioinformatics. 2018 Nov 6;19:403. doi: 10.1186/s12859-018-2426-5 (PMC6219086; doi:10.1186/s12859-018-2426-5)
Supplement: Supplementary file 5 — Files of the example presented in Additional file 4. This additional file is a zip package that contains all files used in Additional file 4. Detailed descriptions of each of these files are presented in a README file included in this zip package. (GZ 30411 kb) [file 12859_2018_2426_MOESM5_ESM.gz › SyNDITools/Meme2Fimo/meme_4.9.0_4.tar.gz!/meme_4.9.0/doc/fasta-subsample.html]

fasta-subsample


# fasta-subsample

**Usage:** `fasta-subsample <fasta> <n> [options]`

**Description:**

Create a random subset of the sequences in a FASTA formatted file. The random seed is fixed
so the same subset will be ouput in every run of the program unless it is explicitly set.

**Input:**

Takes a FASTA file <fasta> and the count of sequences to randomly select <n>.

**Options:**

-seed <random seed>
:   seed the random number generator uses to select the sequences; default: 1

-rest <file>
:   name of the file to send the sequences not selected in the output; default: none

-off <offset>
:   the offset within each sequence to print; default: 1 (no offset)

-len <len>
:   the maxiumum length that printed sequences are constrained to; default: print entire sequence

**Output:**

Writes a FASTA formatted file to standard out containing the specified subsample of the original file.
If -rest <file> is specifed then any left over sequences are written to <file>, which is useful
for cross-validation.
